# Anchorage Accurately Assembles Anchor-Flanked Synthetic Long Reads

**DOI:** 10.4230/LIPIcs.WABI.2024.22

**Published:** 2024-08-26

**Authors:** Xiaofei Carl Zang, Xiang Li, Kyle Metcalfe, Tuval Ben-Yehezkel, Ryan Kelley, Mingfu Shao

**Affiliations:** Huck Institutes of the Life Sciences, The Pennsylvania State University, University Park, PA, USA; Department of Computer Science and Engineering, The Pennsylvania State University, University Park, PA, USA; Element Biosciences, San Diego, CA, USA; Element Biosciences, San Diego, CA, USA; Element Biosciences, San Diego, CA, USA; Huck Institutes of the Life Sciences, The Pennsylvania State University, University Park, PA, USA Department of Computer Science and Engineering, The Pennsylvania State University, University Park, PA, USA

**Keywords:** Genome assembly, de Bruijn graph, synthetic long reads, anchor-guided assembly, LoopSeq, Applied computing → Molecular sequence analysis

## Abstract

Modern sequencing technologies allow for the addition of short-sequence tags, known as anchors, to both ends of a captured molecule. Anchors are useful in assembling the full-length sequence of a captured molecule as they can be used to accurately determine the endpoints. One representative of such anchor-enabled technology is LoopSeq Solo, a synthetic long read (SLR) sequencing protocol. LoopSeq Solo also achieves ultra-high sequencing depth and high purity of short reads covering the entire captured molecule. Despite the availability of many assembly methods, constructing full-length sequence from these anchor-enabled, ultra-high coverage sequencing data remains challenging due to the complexity of the underlying assembly graphs and the lack of specific algorithms leveraging anchors. We present Anchorage, a novel assembler that performs anchor-guided assembly for ultra-high-depth sequencing data. Anchorage starts with a kmer-based approach for precise estimation of molecule lengths. It then formulates the assembly problem as finding an optimal path that connects the two nodes determined by anchors in the underlying compact de Bruijn graph. The optimality is defined as maximizing the weight of the smallest node while matching the estimated sequence length. Anchorage uses a modified dynamic programming algorithm to efficiently find the optimal path. Through both simulations and real data, we show that Anchorage outperforms existing assembly methods, particularly in the presence of sequencing artifacts. Anchorage fills the gap in assembling anchor-enabled data. We anticipate its broad use as anchor-enabled sequencing technologies become prevalent. Anchorage is freely available at https://github.com/Shao-Group/anchorage; the scripts and documents that can reproduce all experiments in this manuscript are available at https://github.com/Shao-Group/anchorage-test.

## 1 Introduction

Sequence assembly has long been a critical task in computational biology, serving as a foundational step in understanding genomic structures and functions. Assembly algorithms have been driven by the rapid evolution of sequencing technologies. Early approaches focused on Sanger sequencing data, requiring algorithms that could handle relatively low-throughput, high-accuracy reads. The advent of next-generation sequencing (NGS) technologies introduced a new era of high-throughput sequencing, producing short reads with low error rates that necessitated the development of efficient and scalable assembly algorithms. Methods based on de Bruijn graphs (dBGs) were developed, such as SPAdes series [1], Velvet [28], ABySS [3], where assembling full-length sequences is formulated as finding a Eulerian path in the dBG. Recently, third-generation sequencing technologies, such as those from PacBio and Oxford Nanopore, have enabled the production of long reads, which, despite higher error rates, provide crucial information for resolving complex genomic regions and structural variants. This has led to the creation of overlap-layout-consensus assemblers, such as Flye series [17], Canu [18], and hifiasm [6]. As sequencing technologies continue to advance, assembly algorithms must continuously adapt, incorporating new strategies and algorithms to take advantage of the new features in the data and to keep pace with the increasing data complexity.

Recent advancements of synthetic long read (SLR) sequencing technologies are able to label reads from the same molecule with the same barcode/index [13, 14, 25]. One representative of such technology is LoopSeq Solo, where exactly one molecule is captured in each plate well. LoopSeq Solo distributes molecular index to every read that evenly covers the entire (long) molecule, whereafter accurate paired-end short reads can be sequenced by any standard next-generation sequencing technology [25, 21]. LoopSeq Solo exhibits high purity of their read clouds, meaning that almost all reads with the same index originated from the same molecule [22]. Taking advantage of its high purity, we are able to assemble each molecule separately instead of assembling a read cloud of multiple molecules. This practice is especially beneficial when the sequenced molecules are similar to each other, such as transcript isoforms and 16S sequences of a microbiome [21].

Modern sequencing technologies can also ligate short adapters with known sequences to both ends of a captured molecule [12, 21, 23]. Those adapters often play functional roles in sequencing, for example, the capture of target molecules, template switch and amplification in PCR [23, 21, 25]. Part of LoopSeq’s adapters are called anchors, which are short synthetic sequences of 12 base pairs and are dissimilar from the sequenced target. Anchors, and likewise, adapters in other sequencing technologies, are extremely useful for assembly as they mark the two endpoints of molecules [24, 23, 21]. We argue that, having the ends of molecules accurately determined transforms the assembly problem into finding a path that connects the ends in an assembly graph. This scheme is computationally easier than finding either Euler path or Hamiltonian path. Previous studies, such as ref [29, 20, 16, 10], have leveraged the edge of a similar idea in connecting both ends of a read pair in an assembly graph and ref [24] showcased end-guided assembly of transcripts with genome reference, but to our best knowledge, such formulation has not been explored in *de novo* assembly of a full-length single molecule with high sequencing coverage. Sequencing depths are crucial for accurate assembly with high-coverage generally preferred. LoopSeq Solo, for example, can produce ultra-high sequencing depth, e.g., more than 1 million reads per molecule. The whole target molecule is therefore sequenced in full coverage without a gap. However, high depth may cause sequencing artifacts, which increases the complexity of the assembly graphs, requiring robust assembly algorithms to fully utilize the high sequencing depth.

Even though assembly has been extensively studied and many assemblers have been developed, none of them is specifically designed for anchor-equipped, ultra-high-depth sequencing data. In this paper we present a new assembler, Anchorage, to fill the gap. Anchorage features a new formulation for assembling anchor-enabled data, that to seek a path in the underlying assembly graph that connects the start/end anchor nodes identified by mapping anchor sequences. Leveraging the high sequencing depth, we seek the path whose minimized node weight is maximized. We design an efficient dynamic programming to find the optimal connecting path. Additionally, Anchorage includes a novel method to estimate the length of the captured sequence. The dynamic programming is adapted to incorporate the estimated length as a selection criterion. Through both simulations and real biological data, we demonstrate that Anchorage outperforms existing assembly methods on anchor-enabled, ultra-high-depth sequencing data. Notably, when sequencing artifacts are present, Anchorage exhibits a significant performance advantage.

## 2 Methods

Anchorage takes reads with the same index, which are known to originate from the target molecule, and the associated anchor sequences, which mark the ends of the target molecule, as input, and assembles the full-length sequence of the target molecule. In its first module, it estimates the length of the target molecule based on frequencies of kmers. Its second module first constructs a compact de Bruijn graph (cdBG) from the raw reads and then searches for candidate paths guided by the anchor sequences. Lastly, the candidate paths will be examined against the range of the estimated target length so as to pick one path, forming the assembled molecule.

### 2.1 Estimating target length

An accurate estimation of the target length is critical for determining the correct target sequence. The LoopSeq data exhibits desirable properties, including high purity (i.e., nearly all reads of a run come from the target molecule), high and evenly distributed coverage, and a low error rate. Many previous studies proposed kmer-frequency-based methods to accurately estimate the size of a genome [15, 26]. In this work, we proposed a new method to estimate the target length accurately by leveraging these properties of LoopSeq.

Let M be the (unknown) length of the target molecule, N be the number of reads, and R be the length of each read. If we assume that reads are uniformly sampled from the target molecule, the target molecule does not contain repetitive kmers, and all reads are error-free, then the frequency of each kmer can be calculated as

$$F_{kmer}=N\times (R-k+1)/M,$$

leading to

$$M=N\times (R-k+1)/F_{kmer}.$$

In practice, N and R are known statistics. The choice of k should ensure that most kmers in the target sequence are unique, i.e., k cannot be too small, while also making most kmers error-free, i.e., k cannot be too large. In our method, we choose k=33 as a default value, which balances well these two considerations for SLR sequencing data. The accurate estimation of M now depends on a “good” estimation of kmer frequency $F_{\text{kmer}}$. The distribution of kmer frequency can be calculated from the sequencing reads. We found that using the average, mode, or median of the frequencies is ineffective, as these statistics are prone to disturbances from sequencing errors and repetitive kmers.

We propose using the *N50 kmer frequency*, defined as the frequency for which the collection of all kmers of that frequency or higher accounts for at least 50% of the occurrences of all kmers. This concept is similar to the N50 of contig lengths, which is known to be more robust against long-tail distributions or local maxima in the frequencies. Although N50 is commonly used as a measure in evaluating genome assembly methods, using the N50 kmer frequency to estimate the target length is a novel approach. By substituting the unknown variable $F_{\text{kmer}}$ with its estimator, N50 kmer frequency, denoted as $F_{\text{N}50k}$, the length of the target molecule M can be easily computed by $M=N\times (R-k+1)/F_{N50k}$. Anchorage also sets upper and lower bounds for the target length (default: 50% and 200% of the estimated target length M). These bounds are later used to choose the best full-length sequence.

### 2.2 Anchor-guided assembly

In addition to cell barcodes and unique molecular index (UMI), modern sequencing technologies can ligate additional known short sequences to both ends of a captured molecule. These short sequences play important roles, such as in template switching and preamplification [23], but also serve as indicators of the endpoints of the target molecule [21, 24]. LoopSeq employs similar short sequences, known as “anchors” [21]. The sequences of the start/end anchors can be mapped to the underlying assembly graph (i.e., a compacted de Bruijn graph in Anchorage) to locate the start/end anchor nodes. The task of assembling the full-length molecule now becomes finding a path from the start anchor node to the end anchor node in the assembly graph. We refer to this task as *anchor-guided assembly*. Note that the search space of anchor-guided assembly is much smaller than searching for the best Eulerian path or Hamiltonian path in the classic assembly formulation, thanks to the critical information provided by the anchors.

We use compacted de Bruijn graph (cdBG) as the assembly graph to organize reads originating from a target molecule (i.e., reads with the same index). In the implementation of Anchorage, SPAdes [1] is called to construct the de Bruijn graph (dBG). In a node-centric dBG, a node represents a distinct kmer and its weight is equal to the number of appearance in the reads. The cdBG is constructed by concatenating each simple path of the dBG as a single node (called a unitig). Each node v∈V has a weight w(v) calculated as the coverage of the unitig v.

Given a weighted cdBG G=(V,E,w) and the anchor sequences, Anchorage starts with identifying start/end anchor nodes by aligning the anchor sequences to the nodes of G. Note that neither the anchor sequences nor the unitigs may be error-free. To be able to tolerate such errors, Anchorage employs an iterative approach: it first locates nodes that can exactly match the anchor sequences (i.e., assuming no errors); if such start/end anchor nodes can be identified and “appropriate” path (i.e., full-length molecule) can be assembled, then the algorithm terminates; otherwise Anchorage increments the tolerance of edit distance by 1 and repeats the procedure, until a user-defined maximum edit distance is reached (default: 2). Since anchor sequences are usually much shorter than the kmer size of a dBG (for instance, anchors of LoopSeq Solo have 12 base pairs), tolerating a maximal edit distance of 2 should be sufficient to locate anchors.

Note also that multiple start/end anchor nodes might be identified in each iteration, due to repeats or sequencing errors/artifacts. Anchorage will consider each pair of start/end anchor nodes and seek an optimal path that connects them in the cdBG. The optimal connecting paths will also be scored, and the one with the maximum score (across all pairs) will be selected and the corresponding full-length sequence will be reported. In case of multiple connecting paths have the same maximum score, the estimated sequence length will be used to break the tie by picking the one whose length is the closest to the estimation. The framework of Anchorage is given as the pseudo-code in Algorithm 1; the algorithm for finding the optimal connecting path together with its score is described in the next section.

**Algorithm 1:** Anchor-guided Assembly.

1:	Input: weighted cdBG G=(V,E,w), start/end anchor sequences $a_{s}$ and $a_{t}$, estimated sequence length M
2:	Output: full-length sequence of the target molecule
3:	**for** e=0→max_tolerated_edit_distance **do**
4:	identify S⊆V where each s∈S contains a substring $s^{\prime }$ such that $d\left(s^{\prime },a_{s}\right)\leq e$
5:	identify T⊆V where each t∈T contains a substring $t^{\prime }$ such that $d\left(t^{\prime },a_{t}\right)\leq e$
6:	let $p^{*}$ be the best path (so far) with score $z^{*}=0$ and length $L^{*}=0$
7:	**for** each pair in {(s,t)∣s∈S,t∈T} **do**
8:	(p,z,L)←connect(G,s,T)
9:	**if** $z>z^{*}$ **or** ($z=z^{*}$ **and** $|L-M|<\left|L^{*}-M\right|$) **then**
10:	$p^{*}\leftarrow p$, $z^{*}\leftarrow z$, $L^{*}\leftarrow L$
11:	**end if**
12:	**end for**
13:	**if** $p^{*}$ is not empty (i.e., $z^{*}>0$ or $L^{*}>0$) **then**
14:	read out the sequence following $p^{*}$ and **return** it (the algorithm terminates)
15:	**end if**
16:	**end for**

### 2.3 Finding optimal connecting path

Let G, s, and t be the given cdBG and the start/end anchor nodes. We aim to find the “optimal” path in G from s to t. We define the optimal path first. Note that the “true” path corresponds to the target sequence, which must be covered by most reads. We therefore define the optimal connecting path to be the one whose smallest node weight is maximized. When there exist two paths whose smallest (node) weight is equally maximized, we compare their second smallest weight, and so on. Formally, let $p_{1}$ and $p_{2}$ be two paths in G from s to t. Let $w_{1}^{i}$ and $w_{2}^{i}$ be the ith smallest weight in path $p_{1}$ and $p_{2}$, respectively. We then define $p_{1}$ to be better than $p_{2}$, if there exists an integer k such that $w_{1}^{i}=w_{2}^{i}$ for all 1≤i<k, and $w_{1}^{k}>w_{2}^{k}$. We argue that this definition is suitable for anchor-guided assembly, as it selects the path with the strongest support from reads, while also automatically ruling out false paths due to sequencing errors which often have low coverage. We note that a similar formulation has been used in the context of reconstructing the entire fragment (or its alignment) of paired-end RNA-seq reads and in transcript assembly [29, 27, 20].

The optimal connecting path can be calculated efficiently using a dynamic programming algorithm, as this definition satisfies the optimal substructure property. Specifically, we define d(l,v) as the maximized smallest weight from s to node v using up to l edges, v∈V. We have this recurrence: $d(l,v)=\max\{d(l-1,v),\max_{(u,v)\in E}(\min(d(l-1,u),w(v))\}$. However, the length of this single optimal sequence may not fall in the reasonable range $\left[M_{l},M_{u}\right]$ (default: $M_{l}=0.5M$, $M_{u}=2M$]). To take into account the estimated sequence length, Anchorage calculates the best c optimal paths, where c is a user-defined parameter (default: c=30). These top c optimal paths can be calculated by extending the above dynamic programming algorithm. Specifically, we replace d(l,v) with a priority queue pq(l,v) of size up to c, storing the maximized smallest weight of the best c paths from s to v using at most l edges. To update, we consider each in-edge (u,v)∈E of v, and examine each element z stored in pq(l−1,u). Let $z^{\prime }=\min\{z,w(v)\}$. If pq(l,v) is full and $z^{\prime }>pq(l,v).\text{smallest-key}()$, which means the examined path to u expanded by (u,v) leads to a better path than the worst one stored in pq(l,v), we update it by doing pq(l,v)⋅pop() and $pq(l,v).\text{insert}\left(z^{\prime }\right)$; if pq(l,v) is not full, we simply do $pq(l,v).\text{insert}\left(z^{\prime }\right)$. This operation takes Θ(c⋅logc) time, Hence, updating all in-edges of all vertices takes Θ(c⋅logc⋅|E|) time.

Note that if c=1 then l can be limited to |V|−1, as the single optimal path must not contain cycles. However, when c>1, paths might contain cycles. Hence, we have to consider l from 1 all the way to |E|, which slows down the algorithm. We can leverage the estimated upper bound $M_{u}$ to speed up. Once a path reaches the upper bound, we can exclude it from expanding. Specifically, an element in a priority queue is now a pair (z,L) where z remains the smallest weight and L stores the corresponding sequence length. The above updating procedure will be executed only if $L+L(u,v)\leq M_{u}$, where L(u,v) denotes the length increased by expanding edge (u,v). Doing so will accelerate the termination, as after certain rounds, which is likely much smaller than |E|, the optimal c paths will not get better, and then the algorithm will (safely) terminate (lines 20–22). The runtime of this algorithm is $\text{Θ}\left(c\cdot \log\;c\cdot |E|\cdot l^{*}\right)$, where $l^{*}$ is the number of rounds executed, $l^{*}\leq |E|$. The space taken by this algorithm is $O\left(c\cdot l^{*}\cdot |V|\right)$ which is the size of the dynamic programming table. The dynamic programming algorithm is given as the pseudo-code in Algorithm 2.

## 3 Results

The anchor-enabled, ultra-high coverage sequencing technology represented by LoopSeq Solo offers an unprecedented opportunity for accurately detecting full-length captured molecules. The high purity of reads allows for assembling each molecule separately, the anchors enable precise determination of endpoints and the high coverage reveals the true molecule as the most abundant path in the assembly graph. All of these superior properties have been leveraged by and modeled in Anchorage. However, these advantages come with some costs. Sequencing artifacts may occur, resulting in more complicated assembly graphs. In Section 3.1, we investigate the sequencing artifacts on 7 real datasets produced by LoopSeq Solo, proving their presence; on the same dataset, we show that the N50 kmer frequency calculated in Section 2.1 gives a more accurate estimation than other methods. We then compare the assembly accuracy of Anchorage with the state-of-the-art assembler SPAdes and its variant on these real data in Section 3.1, on simulated data without artifacts in Section 3.3, and on simulated data with two types of artifacts in Section 3.4 and 3.5.

**Algorithm 2:** Connect a pair of start/end anchor nodes.

1:	Input: graph G=(V,E,w), start/end anchor nodes s and t, length range $\left[M_{l},M_{u}\right]$
2:	Output: an optimal connecting path p from s to t in G with score z and length L
3:	**for** l=1→|E| **do**
4:	**for** each node v∈V **do**
5:	**for** each edge (u,v)∈E **do**
6:	**for** each element (z,L)∈pq(l−1,u) **do**
7:	**if** $L+L(u,v)>M_{u}$ **then**
8:	**continue**
9:	**end if**
10:	$z^{\prime }\leftarrow \min\{z,w(v)\}$
11:	**if** pq(l,v) is not full **then**
12:	$pq(l,v).insert\left(z^{\prime },L+L(u,v)\right)$
13:	**else if** $z^{\prime }>pq(l,v)\text{.smallest-key()}$ **then**
14:	pq(l,v).pop())
15:	$pq(l,v).insert\left(z^{\prime },L+L(u,v)\right)$
16:	**end if**
17:	**end for**
18:	**end for**
19:	**end for**
20:	**if** none of the priority queues gets updated **then**
21:	let $l^{*}=l$ and **break**
22:	**end if**
23:	**end for**
24:	filter out elements (z,L) in $pq\left(l^{*},t\right)$ with $L<M_{l}$
25:	find the element (z,L) in $pq\left(l^{*},t\right)$ with maximized z
26:	trace back for this element to get the optimal path p from s to t
27:	**return** (p,z,L)

### 3.1 Investigation of sequencing artifacts and depths

To investigate the presence of artifacts, we retrieved LoopSeq Solo sequencing reads of seven controlled 16S molecules whose ground truth nucleotide sequences are known (Table 1). Bacterial genomic DNA materials were retrieved from ATCC (catalog number 19718D-5, 47085D-5, BAA-3050, 27774D-5). Ground truth 16S DNA sequences were downloaded from the ATCC genome portal [2]. We first performed quality control using Trimmomatic [4]. Then, reads with the same molecular index were aggregated and their index sequences were trimmed. Afterward, we aligned all the reads to the ground truth sequences using STAR [7], enabling chimera detection with –chimSegmentMin 20. Chimeric reads were analyzed to reveal sequencing artifacts. See the results in Table 1.

We observed 239 to 573 uniquely mapped read pairs supporting back-splicing junctions (BSJs) of 100bp or longer (#BSJs in Table 1). A BSJ is a special type of junction where the donor site is downstream of its acceptor site, opposite to ordinary forward splicing junctions. The BSJs indicate read-through events in the circular amplicon sequencing used by LoopSeq [25], resulting in read-through reads that span the end and the start of the captured molecule. It is noteworthy that the reported reads with BSJs may greatly underestimate the number of read-through reads because Trimmomatic was applied, which trimmed away low-quality regions and read-through reads. Additionally, only properly aligned read pairs, that were reported by the STAR aligner in the SAM format, are included. STAR reported 7.63% to 25.28% unaligned reads, which we believe includes a significant portion of read-through reads as well.

We also observed that approximately 1% of the read pairs from each control formed chimeras between two 16S molecule species (#IMJs in Table 1). Furthermore, six of the seven controls have at least one forward splice junction supported by at least 1,000 uniquely mapped reads. These splice junctions indicate potential artifact molecules. One possible reason for the high IMJ rate is that recombination is more likely to happen between highly similar molecules, e.g. 16S sequences, and the recombinant was formed and amplified in the PCR cycles.

The observed sequencing artifacts significantly increase the complexity of the underlying assembly graph. For example, artifact forward splice junctions result in erroneous edges, inter-molecular chimeras can create both erroneous edges and additional anchor nodes, and intra-molecular back-splice junctions may introduce cycles in the graph. These factors must be considered for a comprehensive comparison of different methods. Therefore, we performed the evaluation on simulated data both with and without the introduction of sequencing artifacts (Sections 3.3, 3.4, and 3.5).

As illustrated in Section 2.1, an accurate estimation of target length is reliant on the accurate estimation of a “good” kmer frequency $F_{kmer}$. We compared N50 kmer frequency with other estimators of $F_{kmer}$, such as the average, median, and mode of kmer frequencies (Table 2). Notably, some estimators were computed based on the frequencies of those kmers whose frequency is higher than 10 or 100, respectively, to rule out fortuitous kmers due to random sequencing errors. Actually, the median of all kmer frequencies is always 1 for all seven controls. Also, estimators based on kmers with a frequency higher than 10 are extremely unreliable and always much worse than those based on kmers with a frequency higher than 100. The N50 kmer frequency is the best estimator of $F_{kmer}$ in six controls and its difference from the ground truth is less than or equal to 10% in five controls, marking its superiority and reliability in application. Even though the mode of frequencies of kmers whose frequency is higher than 100 is the best in the fifth control, the N50 kmer frequency has a very close performance. Nevertheless, it is intriguing and arbitrary to determine under which frequency (in this example, 100) kmers are unreliable. The differences between estimations and ground truths are relatively large in the second and fourth control, this might indicate those two samples have more sequencing errors and artifacts, while N50 kmer frequency is a significantly better and more robust estimator than the other options.

### 3.2 Assembly of real biological samples by LoopSeq Solo

We evaluate the assembly accuracy on the same dataset with seven LoopSeq Solo samples studied in Section 3.1. Reads with the same index were grouped together and piped to each algorithm. Each control has 0.7M–1.9M paired-end reads (Table 1). However, assembling a ~1500bp sequence (length of the known ground-truth molecule) with more than half a million reads is unnecessary, as the tremendously excessive reads (approximately 112,000–304,000× depth) do not contribute new information but errors and artifacts. To fairly reduce time and computational complexity, we sampled 10,000 reads for each control and performed the experiments. This is approximately equal to 1,600× depth which is already ultra-high compared to an ordinary sequence assembly and fits the purpose of this study.

**Figure 1:**
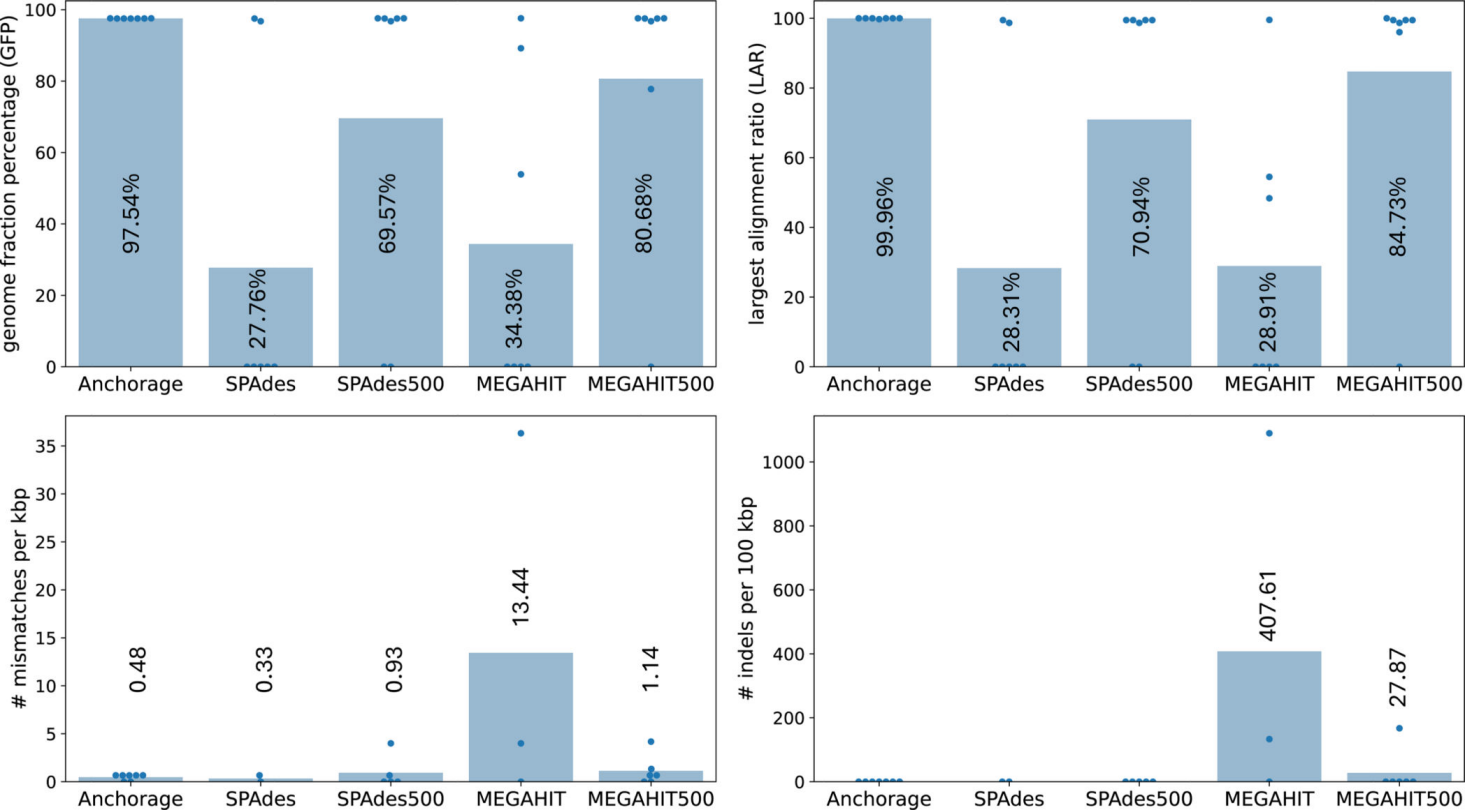
Comparison of assembly accuracy on real LoopSeq Solo sequencing datasets. Anchorage, SPAdes, and MEGAHIT used all reads; SPAdes500 and MEGAHIT500 used 500 reads via random downsampling. The height of each bar represents the average value of each metric and the average value is labeled on each bar. Each dot represents the value of one assembly.

We compared Anchorage with SPAdes [1] and MEGAHIT [19]. Both tools are very popular in assembling single-cell sequencing and metagenomic sequencing reads. Besides, SPAdes is the only assembler that was previously used by and integrated in the LoopSeq analysis pipeline [5, 21]. Since the ultra-high sequencing depth often brings detrimental artifacts to other assembly algorithms (discussed below), we also ran SPAdes and MEGAHIT with randomly down-sampled 500 reads. It is often possible that a state-of-the-art algorithm outputs multiple contigs for one instance. We selected the best contig with the following preference: contigs with both start and end anchors present, contigs with either anchor present, contigs without anchors present, and then trimmed extra sequences outside anchors. If multiple contigs have the same maximal number of anchors identified, the longest contig is selected as the output. Those selection criteria for SPAdes and MEGAHIT follow the current published LoopSeq pipeline [21]. We used QUAST [11] to evaluate the seven assemblies of each algorithm against their respective known ground-truth sequence. The minimal contig length was set to 200 bp. Four metrics are reported (see below). Experimental details such as the parameters used can be found in online code repositories (Section 5). The comparison is given in Figure 1.

QUAST aligns the assembled sequence to the reference (i.e., ground-truth sequence). The first metric is *genome fraction percentage (GFP)*, defined as the percentage of bases in the reference that are aligned by the assembly. This metric reflects the sensitivity of assembly methods. Anchorage achieved a very high GFP for all assemblies, averaging 97.5%. This is 21% and 39% higher than that of the second and third-best methods, MEGAHIT with down-sampled 500 reads and SPAdes with down-sampled 500 reads. Notably, the GFP of SPAdes with all reads is less than half of that of its down-sampled counterpart and the same for MEGAHIT. The second metric is *largest alignment ratio (LAR)*, defined as the ratio of the largest continuous alignment in the assembly, reflecting the precision of different assembly methods. Anchorage achieved a near-perfect LAR for all assembled sequences, 17.8% and 39.4% higher than that of MEGAHIT with down-sampling and SPAdes with down-sampling. Similar to the GFP value, the LARs of SPAdes/MEGAHIT with all reads are less than half of that of their 500-read counterpart. Nevertheless, SPAdes with all reads only assembled two contigs that passed QUAST’s quality filter and properly aligned to the reference. Whilst MEGAHIT with all reads assembled three contigs passing the filter, only one of them has both high GFP and LAR. This might indicate that sequencing artifacts are detrimental to Spades and MEGAHIT with all reads, but down-sampling could help in removing those damages. It is noteworthy that the 2 (resp. 5) assembled sequences by SPAdes with all reads (resp. SPAdes with down-sampling) are very accurate while the other 5 (resp. 2) assembled sequences almost did not align to the reference at all (blue dots in Figure 1).

QUAST also reported the average *number of mismatches per 1000bp* and the average *number of indels per 100kbp*. The rate of mismatches of Anchorage and SPAdes with all reads are very low and no indels were reported in them. On the other hand, MEGAHIT with all reads has the highest mismatch rate and indel rate, at least 10 times higher than the others. QUAST did not report misassemblies on any of the assemblies.

The presence of anchors in the assembled sequence is a positive signal suggesting an assembly is full-length. LoopSeq pipeline, for example, considers an assembled sequence as full-length if it contains both anchors [21]. In this experiment, Anchorage identified both anchors for all 7 sequences (not a surprise, as Anchorage directly models anchors), while the second best method, MEGAHIT with 500 reads, missed the end anchor for one control and missed both anchors for another control. However, the presence of anchors does not always indicate completeness. For example, SPAdes with all reads reported both anchors for 6 of its assemblies but only two of them aligned to their respective ground truth. This observation indicates that even though anchors provide many benefits in an accurate assembly, a sole reliance on anchors to determine the completeness of an assembly is inaccurate.

Anchorage had a reasonable time and memory usage on those real biological datasets, although its running time is the longest among all tools. Anchorage took 77.94s/205.8MB to assemble all seven contigs. SPAdes took 54.67s/205.7MB to assemble with all reads and 42.98s/187Mb to down-sample and assemble with 500 reads. MEGAHIT took 2.00s/208MB to assemble with all reads and 3.37s/205MB to down-sample and assemble with 500 reads. Random down-sampling of reads was performed using Python scripts from the LoopSeq pipeline and it was counted in the total time. All experiments were performed on a 2020-model iMac with 8 Intel i7 Cores and 64 GB Memory.

### 3.3 Assembly of simulated SLR data without artifacts

We then evaluated Anchorage and compared it with other methods on anchor-enabled SLR data with simulations. The ground-truth, full-length sequences were retrieved from NCBI, comprising of 23 16S genes of length ranging from 547bp to 2089bp (see detailed description in Table 3). Subsequently, two 12bp anchors (start: CGCAGAGTACAT, end: TTGGAGTTAAAG), which are the same as used in real LoopSeq Solo sequencing, are concatenated to respectively the start and end of each sequence. Afterward, Polyester [9] was used to simulate 110bp-long paired-end reads with a 0.5% error rate. We simulated a series of sequencing depths of 50×, 100×, 500×, 3000× from ordinary depth to ultra-high depth. Reads from the same 16S gene are grouped together and piped to downstream assemblers, so that the grouped reads can be considered as index-aggregated LoopSeq Solo reads after quality control and trimming. Each method assembles each of the 23 samples into a full-length sequence using the method described in Section 3.2. The assemblies were then evaluated using QUAST [11] against their respective ground truths.

Overall, when no sequencing artifacts are present and the sequencing depths are high, all methods produced accurate assemblies. When depth is higher or equal to 500×, all methods achieved 95% or higher averaged GFP (over the 23 instances). SPAdes and MEGAHIT performed the best when using all reads, but they were closely followed by Anchorage. Anchorage has a lower GFP when the sequencing depth is lower than 100×. This could be because Anchorage models the minimal weight of nodes in a path that is less robust under low depths. The average GFP gradually increases as the sequencing depth increases for both Anchorage and SPAdes with all reads. However, this trend is not observed for methods with down-sampled 500 reads, likely due to missing or decreased coverage in some regions caused by down-sampling. As for LAR metrics, We can see that all methods achieved nearly perfect precision. Anchorage and two MEGAHIT methods reported zero mismatches and indels across all sequencing depths.

### 3.4 Assembly of simulated SLR data with read-throughs

The aforementioned read-through scenarios are more prevalent in high sequencing depth. To evaluate the impact of such a scenario on the assembly methods, we simulated reads with read-throughs. The simulation was done by concatenating an “anchored” 16S gene to itself 5 times so that reads may span from the end of the sequence to its start, stimulating the “read-though” events. The other simulation settings are the same as in Section 3.3.

**Figure 2:**
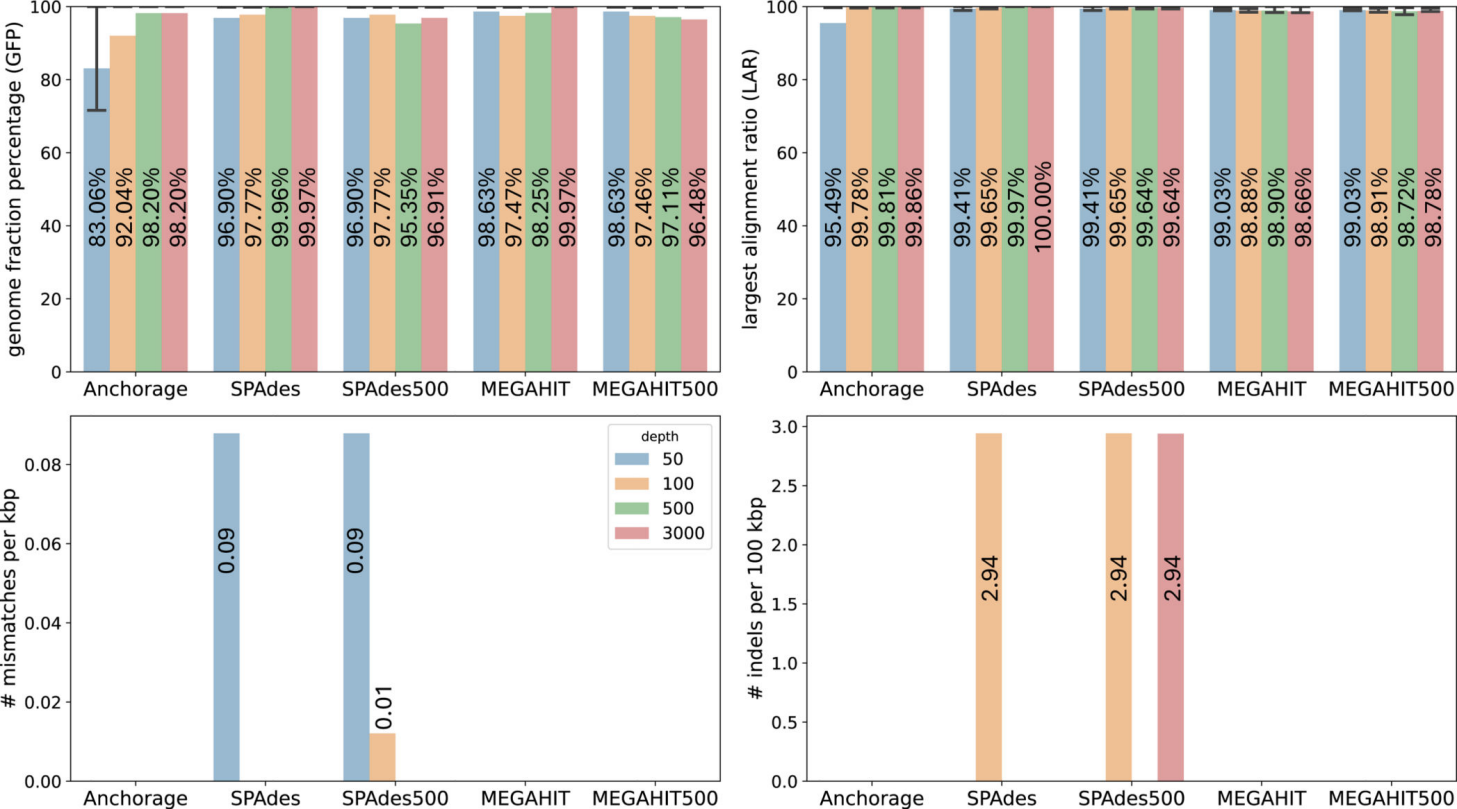
Comparison of assembly accuracy on simulated reads without artifacts. Anchorage, SPAdes, and MEGAHIT used all reads; SPAdes500 and MEGAHIT500 used 500 reads via random downsampling. The height of each bar represents the average value of each metric and the average value is labeled on each bar. The whiskers in the GFP and LAR panels extend from the 25th to 75th percentile of values in each metric.

**Figure 3:**
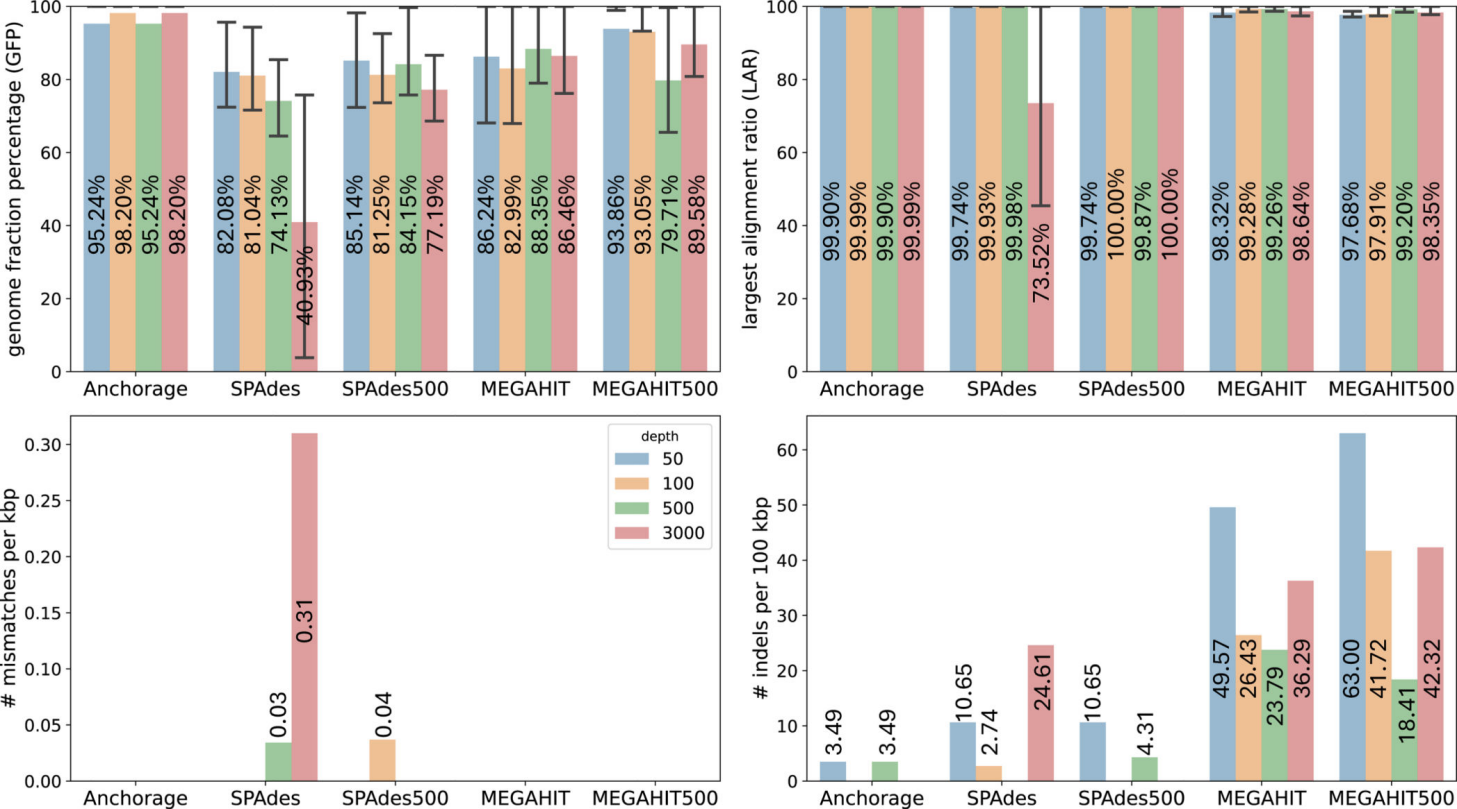
Comparison of assembly accuracy on simulated reads with read-throughs. Anchorage, SPAdes, and MEGAHIT used all reads; SPAdes500 and MEGAHIT500 used 500 reads via random downsampling. The height of each bar represents the average value of each metric and the average value is labeled on each bar. The whiskers in the GFP and LAR panels extend from the 25th to 75th percentile of values in each metric.

The results were demonstrated in Figure 3. Compared to the simulation results without read-throughs (Figure 2), the accuracy of Anchorage was minimally impacted: it achieved a greater than 95% GFP which is better than all other methods on all sequencing depths, and nearly perfect LAR for all sequencing depths. On the contrary, the GFPs of the other methods were much reduced in the presence of read-throughs. The numbers of two MEGAHIT methods and SPAdes with 500 reads dropped to approximately 77–89% from *>*96% under various depths. Furthermore, SPAdes with all reads was impacted the most. Its GFP dropped to 41% from 99% under 3000× depths. Whilst the LARs of all algorithms are almost equally satisfying with being around 99%, SPAdes using all reads had its PAR dropped from 100% to 73.5% under 3000× depths. Those observations confirmed that Anchorage is more robust to sequencing artifacts such as read-throughs, thanks to its design that leverages the anchors to accurately determine the sequence ends. Anchorage and MEGAHIT reported no mismatches in this experiment, but both of the two MEGAHIT methods’ indel rate is very high around 18–63 indels per 100kbp. QUAST reported zero misassemblies for all assemblies.

### 3.5 Assembly of simulated SLR data with repetitive regions

Repeats in the molecule pose a major challenge to assembly methods, as they cause tangled assembly graphs while making the length of the target molecule much harder to estimate. We simulated anchor-enabled SLR data with repetitive regions and read-throughs to test its impact on the assembly methods. For each of the 16S gene used in Section 3.3, we randomly copied 10% of each sequence and inserted it back into themselves at a random location. The other simulation parameters are again the same as previously described in Section 3.3. All simulated data are tested with all methods.

**Figure 4:**
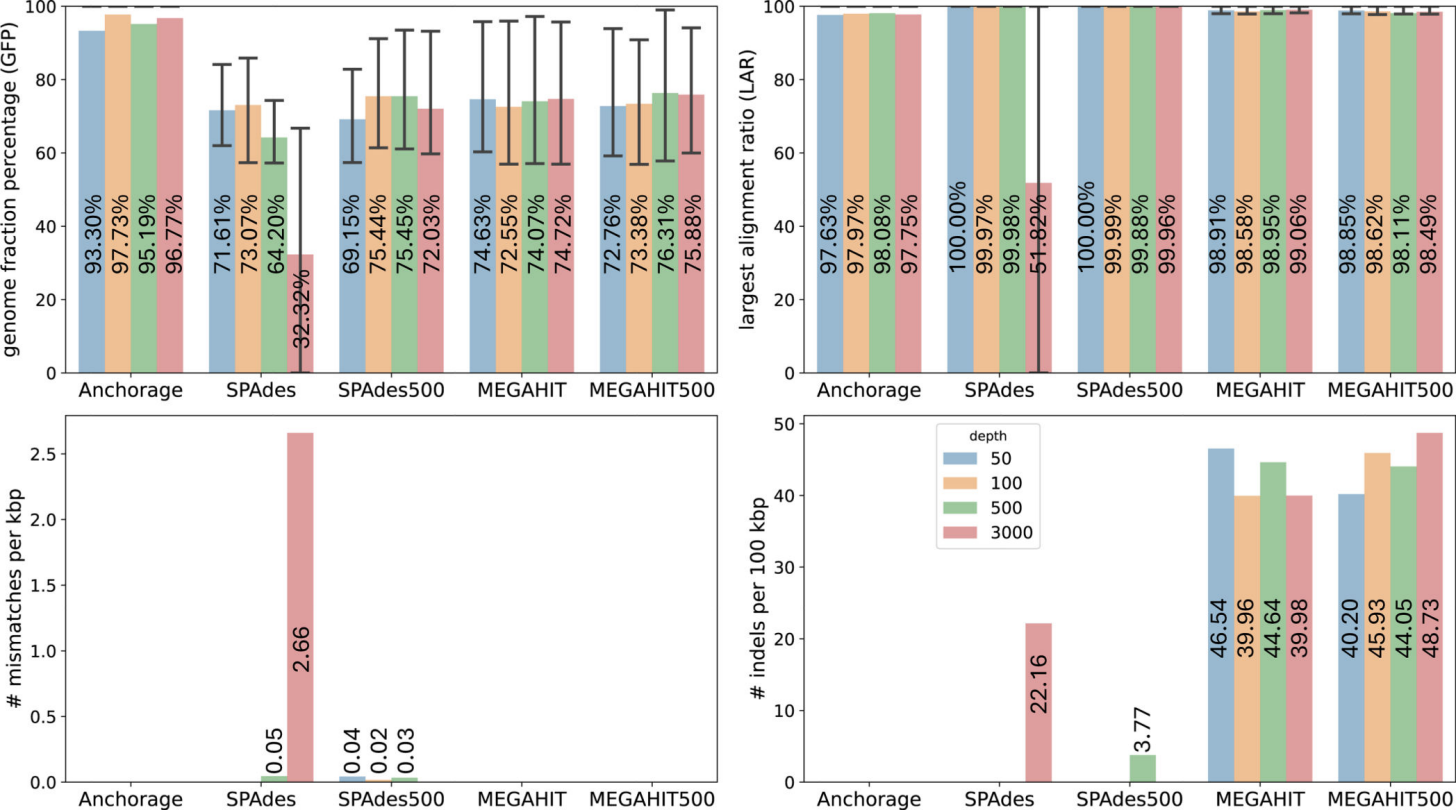
Comparison of assembly accuracy on simulated reads with repetitive sequences. Anchorage, SPAdes, and MEGAHIT used all reads; SPAdes500 and MEGAHIT500 used 500 reads via random downsampling. The height of each bar represents the average value of each metric and the average value is labeled on each bar. The whiskers in the GFP and LAR panels extend from the 25th to 75th percentile of values in each metric.

The comparison was given in Figure 4. The assembly of sequences with repeats appears harder for all methods, evident by the drop in GFP of all methods under all depths. Anchorage achieved the highest GFPs, topping at 96.8%, for all sequencing depths, which is approximately 24%–29.5% higher than the second-best method under various sequencing depths. Unlike previous experiments where GFP of Anchorage increases as the depth increases, assemblies with repetitive regions in different sequencing depths exhibit roughly the same level of accuracy, indicating the source of error is mainly from the complicated structures of the assembly graphs caused by repetitives instead of insufficient coverage. The LARs of most algorithms are near-perfect, with the exception of SPAdes with all reads under 3000×. SPAdes with all reads have a higher mismatch rate while the other methods reported almost zero mismatches. Both two MEGAHIT methods report high indels rates in this experiment. Both Anchorage and MEGAHIT have misassemblies in several contigs under various sequencing depths, most of which are duplications of themselves. This indicates that the read-throughs with repetitive sequences impact both algorithms and more careful algorithm curation is needed. On the other hand, SPAdes with all reads take a more conservative strategy to assemble shorted contigs, as reflected in its low GFP.

## 4 Conclusion and Discussion

We introduce Anchorage, a novel sequence assembler designed for anchor-enabled, high sequencing depth synthetic long reads data. Anchorage incorporates several algorithmic innovations, including a robust k-mer-based method for estimating the length of the target molecule, an innovative approach that efficiently models anchors and high sequencing depth while being resilient to sequencing errors and artifacts, and an efficient dynamic programming algorithm that identifies optimal paths while integrating the estimated sequence length. We evaluated Anchorage against state-of-the-art methods using both simulated and real datasets. Anchorage demonstrated significantly improved accuracy in the presence of sequencing artifacts. Moreover, unlike other methods that experience decreased accuracy with larger input sizes, Anchorage maintains robust and consistent performance, particularly with high sequencing depth.

We would like to note that Anchorage is highly accommodated to assemble anchor-labeled single molecules, where the targeted molecules often have lengths between several hundred to dozens of thousand base pairs, such as RNA transcripts and 16S genes. One major advantage of SLRs, exemplified by LoopSeq, is that reads from a relatively small region are labeled and aggregated prior to assembly. Hence, assemblies of each SLR are separated in a pure read cloud. The SLR assembly task differs from assembling continuous contigs of a whole human-sized genome. Consequently, one single continuous assembly is strongly preferred rather than scaffolds of a large genome. The two state-of-the-art assembly methods, SPAdes and MEGAHIT, are not specifically designed for this task. While they may assemble partial scaffolds, stitching partial assemblies together for a continuous contig requires adequate manual curation and prior knowledge of the sequenced target. We admit that in the case of assembling large genomes, the information provided by anchors and coverage will be diluted and the admixed read clouds increase the problem complexity very much, so Anchorage requires considerable modifications to perform general-purpose genome assembly.

To the best of our knowledge, LoopSeq and LoopSeq Solo are the only sequencing technologies that produce anchor-equipped, high-coverage data. Consequently, these were the only real datasets we tested. However, Anchorage is applicable to any data that possesses these two properties. For example, in principle, adding short synthetic anchors to sequence adapters is practical and increasing sequencing depths requires only increasing PCR amplification cycles. As such sequencing technologies become more prevalent, we anticipate that Anchorage will see broad adoption. As a future direction, we plan to extend Anchorage to assemble multiple target molecules, enabling applications in transcript assembly, metagenome assembly, and synthetic long-read (SLR) assembly with lower purity.

We acknowledge that the dynamic programming algorithm can be further optimized by incorporating ideas from existing algorithms for the *k* shortest paths problem, such as Eppstein’s algorithm [8]. Anchorage currently employs a straightforward algorithm for ease of implementation. To scale for large-scale data in case of need, these advanced optimization techniques can be integrated in the future.

## Figures and Tables

**Table 1:** Information of controlled LoopSeq Solo sequencing of seven 16S molecules. seq length: length of 16S sequence in nucleotides; #read pairs: number of read pairs after quality control; #IMJs: number of inter-molecular junctions; #BSJs: number of back splice junctions; %unaligned: percentage of unaligned reads in all quality-controlled paired-end reads.

No.	Species	seq length	#read pairs	#IMJs	#BSJs	%unaligned
1	*D. desulfuricans*	1546	1896118	20383	519	8.44
2	*N. europaea*	1534	1955927	19310	534	25.28
3	*E. coli*	1538	1956516	20523	521	7.65
4	*N. europaea*	1534	1841484	16128	515	20.44
5	*P. aeruginosa*	1531	1761401	19284	569	8.09
6	*P. aeruginosa*	1531	1610901	16759	494	7.83
7	*E. coli*	1538	717168	7773	235	7.63

**Table 2:** Comparison of different estimators for a “good” kmer frequency. Real: real kmer frequency; N50: N50 kmer frequency; Avg: average; Med: median; kmer freq > 10: computation based on kmers whose frequency is at least 10; kmer freq > 100: computation based on kmers whose frequency is at least 100. Bold estimator is the best in each experiment.

No.	Real	N50	Avg	kmer freq >10			kmer freq >100		
				Avg	Med	Mode	Avg	Med	Mode
		
1	1146	**1050**	21	199	16	12	961	1024	1255
2	1137	**780**	20	179	17	10	579	639	263
3	1127	**1031**	23	213	16	10	889	983	1028
4	1134	**776**	21	161	16	10	563	551	102
5	1131	1035	22	211	16	10	909	1001	**1185**
6	1128	**1016**	23	202	15	10	906	987	959
7	1148	**1040**	27	223	16	10	893	986	1288

**Table 3:** Information of 16S sequenced used in simulation. All species and 16S sequences were selected by searching “16S RefSeq Nucleotide sequence records” (keyword “33175[BioProject] OR 33317[BioProject]”) on NCBI.

Accession #	seq length	Species	Gene name
NR_181961.1	547	*M. ovipneumoniae*	ATCC 29419 strain Y98 16S ribosomal RNA
NR_181928.1	566	*X. bonasiae*	strain FX4 16S ribosomal RNA
NR_178827.1	640	*G. deserti*	strain I12A-02624 16S ribosomal RNA
NR_178392.1	694	*E, entomophila*	strain BR193 16S ribosomal RNA
NR_178393.1	696	*E. nematocerorum*	strain BR208 16S ribosomal RNA
NR_181953.1	740	*A. tiandongensis*	strain 3.1105 16S ribosomal RNA
NR_178227.1	816	*B. bavariensis*	PBi 16S ribosomal RNA
NR_178832.1	830	*X. rhizosphaerae*	strain MH17 16S ribosomal RNA
NR_179969.1	909	*P. piersonii*	strain IIIF1SW-P2 16S ribosomal RNA
NR_180430.1	959	*S. miscanthi*	strain AK13 16S ribosomal RNA
NR_181766.1	1416	*G. fulvus*	strain con5 16S ribosomal RNA
NR_181783.1	1418	*P. piersonii*	strain NRRL B-65522 16S ribosomal RNA
NR_181751.1	1427	*R. ruber*	strain JC435 16S ribosomal RNA
NR_181947.1	1441	*G. pseudamarae*	strain CON9 16S ribosomal RNA
NR_181997.1	1470	*N. flavus*	strain IFO 14396 16S ribosomal RNA
NR_181962.1	1488	*B. bavariensis*	PBi 16S ribosomal RNA
NR_181850.1	1526	*S. parmotrematis*	strain Ptm05 16S ribosomal RNA
NR_181950.1	1538	*E. nematocerorum*	strain BR208 16S ribosomal RNA
NR_181949.1	1538	*E. entomophila*	strain BR193 16S ribosomal RNA
NR_181964.1	1555	*D. oleivorans*	Hxd3 16S ribosomal RNA
NG_044969.1	2089	*T. shockii*	strain WB1 16S ribosomal RNA gene
NG_042068.1	2197	*A. pernix*	culture NBRC:100138 16S ribosomal RNA gene
NG_046384.1	3600	*P. ferrireducens*	strain 1860 16S ribosomal RNA gene
